# Editorial: The roles of social media in education: affective, behavioral, and cognitive dimensions

**DOI:** 10.3389/fpsyg.2023.1287728

**Published:** 2023-09-22

**Authors:** Hung Phu Bui, Mark Bedoya Ulla, Veronico N. Tarrayo, Chien Thang Pham

**Affiliations:** ^1^University of Economics, Ho Chi Minh, Vietnam; ^2^Walailak University, Tha Sala District, Thailand; ^3^University of Santo Tomas, Manila, Philippines; ^4^TNU-University of Sciences, Thái Nguyên, Vietnam

**Keywords:** social media in education, technology-enhanced teaching, technology-aided learning environment, computer-aided learning (CAL), technology-enhanced learning

The interface between education and technology has become both inevitable and significant in today's digitally connected world. As a result, the current educational landscape is shifting toward using digital technologies for teaching and learning (Rautela, 2022). In higher education, for instance, an increasing number of teachers and students use social media for personal and educational purposes (Sabah, 2023). Education is undergoing tremendous modifications across academic disciplines, owing mainly to the integration of social media and web-based platforms (Chau and Bui, 2023). Within this context, educators are pushing boundaries, developing creative approaches, and analyzing outcomes across various teaching and learning situations, from Tencent Docs to Telegram and Instagram to Messenger. This Research Topic explores education in the age of social media, engaging in a discourse where traditional practices meet radical technological needs and trends. It looks deeply into technological shifts, analyzing the promises, successes, and issues that arise from integrating technology, particularly social media, into the ever-changing realm of education. The collected papers in this Research Topic provide a holistic understanding of current educational changes by covering affective, behavioral, and cognitive dimensions (Bui, 2023) and spanning areas such as writing, speaking, and grammar learning, as well as pertinent discussions on physical education, research, professional development, and assessment. Through the eyes of scholars, we examine a range of studies, from experimental interventions and empirical studies to insightful reviews, all with the goal of understanding how the digital age is transforming pedagogical approaches and student experiences.

As educators recognize the pervasiveness of social media in students' lives, research into its integration into English language teaching (ELT) has become critical to identify best practices and evaluate the effectiveness of social media use in supporting language learning. To exemplify, the growing research interest in incorporating social media into ELT highlights its potential to improve writing skills as educators use digital platforms to facilitate authentic writing experiences and immediate peer feedback for learners, as well as increase writing motivation. This Research Topic includes Y. Li's research that examined the impact of online collaborative writing instruction on Chinese English as a foreign language (EFL) students using Tencent Docs, focusing on writing performance, writing self-efficacy, and writing motivation. Out of 58 participants, half used Tencent Docs for tasks outside the classroom (experimental group), while the other half followed traditional in-class instruction (control). Over 13 weeks, the group using Tencent Docs exhibited significantly improved writing performance, motivation, and self-efficacy compared to the control group. In a related research, Zhao and Yang explored the effects of a flipped course on Chinese EFL students' writing performance and anxiety levels using a quasi-experimental approach. Fifty students from two classes were divided into two groups: a traditional instruction group (control) and a flipped instruction group using social media (experimental). Two writing assignments and a writing anxiety scale were used to collect data. The results showed that the experimental group improved significantly at writing and reported less anxiety. Another experimental intervention was conducted in the study of Dai et al., which investigated the impact of wiki-based writing methods on Chinese EFL students' writing skills and self-efficacy. Fifty-three students from a language school in China participated and were divided into two groups: one using the wiki method (experimental) and the other using traditional teaching (control) over three months. Both groups were tested before and after the study using IELTS writing tasks and a writing self-efficacy scale. While both groups showed improvement, those taught using the wiki-based method had more significant gains in writing skills and confidence.

Similarly, scholarly interest in using social media to improve English speaking skills is growing, as its capacity to provide learners with real-world conversational experiences and increased confidence is recognized. Zhou's research explored how online language exchanges affect Chinese postgraduate students' speaking abilities and willingness to communicate (WTC) in an advanced English program. Two groups were compared: one using the Tandem app to converse with foreign English speakers (e-tandem), and the other having collaborative speaking tasks in class (conventional). Fifty-eight students were split between these groups. Data from IELTS speaking tests, a WTC scale, and semi-structured interviews showed that both groups improved their speaking skills. Yet, the e-tandem group excelled more than the conventional group. In a review, Fan delved into how digital-based flipped classrooms influence EFL learners' WTC and self-efficacy. The literature review revealed that social media and digital content can impact students' communication intentions in these classrooms. EFL learners in flipped classrooms demonstrated greater self-efficacy than in traditional settings. The analysis likewise provides insights for EFL educators, educational policymakers, and advisors on enhancing learner self-efficacy, WTC, and the benefits of the flipped learning approach.

Other relevant areas in language education, such as grammar learning, foreign language learning motivation, and the use of the flipped learning approach, were likewise covered in this issue. Teng et al. analyzed the effects of Instagram-feed-based tasks on EFL students' grammar learning. Eighty-four intermediate EFL students were divided into two groups: one received typical online lessons (control), and the other used Instagram-feed-based tasks (experimental). The results, analyzed using one-way ANCOVA, showed that the experimental group learned grammar more effectively than the control group. The findings emphasize the potential of Instagram-feed-based tasks in enhancing grammar learning, and students expressed favorable views toward this method. On the other hand, Zhao et al. investigated the effect of Telegram on foreign language motivation, foreign language anxiety, and learning attitudes of 60 intermediate Iranian EFL students. These students were divided into two groups: one used the Telegram app (experimental), while the other learned traditionally without using social media (control). After 18 sessions, tests revealed that the experimental group had higher motivation, reduced foreign language anxiety, and a positive view of the app's role in their English learning.

In a conceptual review, Pang examined how a web-based flipped learning approach impacts learner engagement and critical thinking. Previous research highlighted the role of social media in fostering these skills and promoting collaborative learning and high-quality interactions, thus boosting student engagement. Furthermore, these platforms offer feedback and complex tasks, honing EFL learners' critical thinking. A corollary to this, Han's review analyzed the flipped classroom approach in language education, particularly its advantages and challenges when integrated with social media. The approach revolves around students accessing lecture content before class, using popular social media platforms for interactive learning. An analysis of 25 journal articles revealed that the flipped approach enhances learning outcomes, including motivation, attitude, course satisfaction, and self-efficacy in higher education. However, a significant challenge is students' unfamiliarity and difficulty adapting to this model. Focusing on an affective dimension in EFL learning, B. Li explored the potential of social networking to boost commitment and dedication in EFL students, providing valuable insights for language educators. By integrating social networking into educational platforms beyond the classroom, the conventional teaching approach is transformed. Social networking, a subset of social media, enables students to interact with peers through online and mobile platforms. This technology fosters a learning environment based on interactive dialog between students.

A few studies and conceptual reviews likewise have delved into the influence and use of social media in other facets of education, such as physical education, research, professional development, and assessment. Wang et al.'s review synthesized previous findings to discuss social media's role in student engagement both in in-class and online sessions. It likewise explored social media's impact on engagement, delved into engagement types, and examined the correlation between social media use and student engagement. In a related review, Chen and Xiao evaluated research on the impact of extensive social media use on students' emotional wellbeing. While positive and negative effects were noted, the latter, including symptoms such as depression, anxiety, and stress, were more prominent. The social comparison theory suggests that several issues stem from students comparing their lives to the unrealistic portrayal of others on social media. Thus, educators, policymakers, and school authorities may be informed about the potential psychological repercussions of pervasive social media use among students.

Moreover, Xu et al.'s work aimed to develop and validate the Social Media Perception Scale for future Physical Education teachers (SMPS-PPE). Data was gathered from 977 preservice physical education teachers using a survey. The data underwent item analysis, exploratory factor analysis, and confirmatory factor analysis. The results indicated that SMPS-PPE is reliable in terms of content validity, internal structure validity, and internal consistency, and valid in evaluating the social media perceptions of these preservice teachers. Lu et al., on the other hand, looked into how novice EFL teachers in the Czech Republic view the use of social media tools, such as Web 2.0, and their willingness to employ them for collaboration in diverse classroom settings. One hundred teachers from various parts of the country participated in a survey and follow-up semi-structured interviews. The results showed that the teachers most open to integrating social Web 2.0 technologies had the most pronounced positive and negative views on them. The level of technology expertise, workload, and work environment influenced these views.

In the area of research, Alonzo and Oo employed autoethnography to analyze their three-year experience using Messenger for collaborative research, discussing the benefits and challenges of utilizing social media for academic collaboration. They showcased how a particular social media tool aided in enhancing their research output and obtaining a grant. The activity theory was used to discuss how various factors (i.e., personal, socio-emotional, structural, technological, and organizational) played a role in the success of their scholarly pursuits. On the other hand, Ping, in a review, explored the influence of teachers' commitment and identity on their use of social media in professional development (PD) for EFL instruction. Social media enhances teachers' dedication and professional identity (PI). Such PD helps teachers envision and shape a new identity through social media interactions. Since identity is fluid, participating in social media communities helps educators collaborate and connect, fostering their PD and professional success.

Lastly, in the area of assessment, Alonzo et al. utilized PRISMA (Preferred Reporting Items for Systematic Reviews and Meta-analyses) to analyze 167 articles on the use of social media in educational assessments, finding only 17 relevant for detailed review. It revealed that Facebook and Twitter were the main platforms for assessment activities, including task sharing, monitoring progress, and offering feedback. The benefits included timely feedback and enhanced student performance. However, concerns emerged about assessment reliability, the constraints of social media tools, and balancing academic with social engagement.

The use of social media and digital platforms in education is no longer a budding trend; it is an essential component of modern pedagogy when harnessed with purpose and prudence. The scholarly works included in this Research Topic show both the transformative power of this integration and its potential challenges. While several educators and students have experienced significant improvements in areas such as writing, speaking, and learning motivation, there are evident concerns, such as the potential psychological consequences of excessive social media use. As the educational world merges with digital technology, educators, policymakers, and stakeholders should create a balanced approach to ensure that the benefits of technology are realized without compromising learners' holistic wellbeing.

## Author contributions

HB: Conceptualization, Writing—review and editing. MU: Conceptualization, Writing—review and editing. VT: Conceptualization, Writing—review and editing. CP: Conceptualization, Writing—review and editing.
